# Treatment-Induced PD-L1 Dynamics and Temporal Heterogeneity in Locally Advanced and Metastatic Gastroesophageal Cancer—A Systematic Review

**DOI:** 10.3390/cancers18183065

**Published:** 2026-09-21

**Authors:** Marharyta Khaustova, Elisabetta Liberanome, Annamaria De Giorgi, Luigi Pio Guerrera, Fausto Petrelli, Gianluca Tomasello, Michele Ghidini

**Affiliations:** 1 Medical Oncology Division, ASST Sette Laghi, 21100 Varese, Italy; margokhaustova28@gmail.com (M.K.); annamaria.degiorgi@asst-settelaghi.it (A.D.G.); luigipio.guerrera@asst-settelaghi.it (L.P.G.); michele.ghidini@uninsubria.it (M.G.); 2 Department of Medicine and Technological Innovation (DiMIT), University of Insubria, 21100 Varese, Italy; eliberanome@studenti.uninsubria.it; 3 Oncology Unit, ASST Bergamo Ovest, 24047 Treviglio, Italy; faupe@libero.it; 4 Oncology Unit, ASST Crema, Largo Dossena 2, 26013 Crema, Italy

**Keywords:** PD-L1, gastric cancer, gastroesophageal cancer, immunotherapy, immune checkpoint inhibitors, tumor heterogeneity, combined positive score, liquid biopsy

## Abstract

**Background/Objectives:** Standard guidelines for treating locally advanced and metastatic gastroesophageal cancer (GEC) involve combination regimens of chemotherapy and immunotherapy. Patient eligibility for these treatments is primarily guided by the programmed cell death ligand 1 (PD-L1) combined positive score (CPS). This approach assumes that a single baseline biopsy accurately reflects a stable, tumor-wide PD-L1 status, an assumption increasingly challenged by spatial and temporal heterogeneity. This systematic review aims to synthesize existing data on PD-L1 expression dynamics before and after systemic therapy in GEC, and to evaluate whether these changes differ between neoadjuvant and metastatic settings. **Methods:** We systematically searched PubMed/MEDLINE (up to May 2026) for studies reporting paired pre- and post-treatment PD-L1 status in gastric and gastroesophageal junction cancers. This systematic review was conducted in accordance with the PRISMA 2020 guidelines. Narrative synthesis and qualitative data transformation were structured following the Synthesis Without Meta-analysis (SWiM) reporting framework. Study-level risk of bias was assessed using the Newcastle-Ottawa Scale (NOS). **Results**: Eleven studies were included in this systematic review, stratified into neoadjuvant (*n* = 6) and metastatic (*n* = 5) settings. Our findings suggest that changes in PD-L1 expression in gastroesophageal cancer depend heavily on the clinical setting. Specifically, an upward trend in post-treatment PD-L1 expression was evident in the neoadjuvant setting, with 5 of 6 studies reporting a prevalence of PD-L1 gain ranging from 6.3% to 33.3%. Conversely, PD-L1 downregulation was consistently reported across all metastatic studies, with loss rates ranging from 19.3% to 44.4%. **Conclusions:** PD-L1 status in gastroesophageal cancer changes dynamically after treatment. These findings highlight the limitations of relying on archival tissue and support the use of repeat biopsies to track evolving tumor phenotypes. This study is primarily limited by small sample sizes of paired biopsies, technical heterogeneity in PD-L1 scoring and treatment regimens, potential publication bias, and the lack of prospective registration.

## Simple Summary

Immunotherapy for gastric and esophageal cancers largely relies on a single tumor biopsy that assesses PD-L1 expression. This approach assumes that PD-L1 levels remain stable throughout the tumor and over time. However, growing evidence suggests that its expression can dynamically change during treatment. In this systematic review, we analyzed 11 studies involving 1025 patients and 621 paired samples to evaluate PD-L1 expression patterns before and after therapy. We observed that PD-L1 levels tended to increase after neoadjuvant treatment in localized disease, whereas in metastatic disease, changes were more frequently downregulated. These findings suggest that a single, baseline biopsy may misclassify patient eligibility for immunotherapy. Consequently, repeat testing during treatment via re-biopsy or blood-based liquid biopsy could improve clinical decision-making and better tailor personalized therapy.

## 1. Introduction

Gastroesophageal cancers (GECs) remain a major therapeutic challenge, with high mortality rates and limited treatment options for advanced disease. According to GLOBOCAN 2022 estimates, esophageal cancer accounted for 511,054 new diagnoses, while gastric cancer contributed another 968,784 cases. Together, these 1,479,838 new cases represented approximately 7.4% of all global cancer diagnoses [1].

Over the past decade, the management of GECs shifted considerably with the introduction of immune checkpoint inhibitors targeting the PD-1/PD-L1 pathway [2,3]. For perioperative settings, FLOT chemotherapy was long considered the standard of care for resectable cases; however, the phase III MATTERHORN trial recently demonstrated the benefit of adding durvalumab to this regimen [4,5]. Durvalumab was introduced directly into the perioperative setting, with pCR rates of 19.2% [95% CI, 15.7–23.0] vs. 7.2% [95% CI, 5.0–9.9] in the control group. Furthermore, it demonstrated a significant improvement in both EFS (median NE [95% CI, 40.7–NE] vs. 32.8 months [95% CI, 27.9–NE]; HR 0.71 [95% CI, 0.58–0.86]; *p* < 0.001) and OS (NE vs. NE; HR 0.78 [95% CI, 0.63–0.96]; *p* = 0.021) in the ITT population, revolutionizing resectable GC care [4].

For the metastatic cohort, the CheckMate-649 trial opened the perspective of ICIs for the first line in HER2-negative GC, introducing nivolumab to standard chemotherapy (FOLFOX/XELOX) (HR 0.71 [98.4% CI, 0.59–0.86]; *p* < 0.0001) and the KEYNOTE-811 trial in HER2-positive GC added pembrolizumab to trastuzumab plus chemotherapy regimen (median OS of 20.0 vs. 16.8 months; HR 0.80; [95% CI, 0.67–0.94]; *p* = 0.0040) [2,6]. In 2023 the KEYNOTE-859 trial established the effectiveness of combining first-line pembrolizumab with chemotherapy in patients with HER2-negative gastric cancer, showing a statistically significant increase in median overall survival (12.9 months [95% CI, 11.9–14.0] as compared with 11.5 months [95% CI, 10.6–12.1]; HR 0.78 [95% CI, 0.70–0.87]; *p* < 0.0001) [3]. The RATIONALE-305 study also provided further evidence of a statistically significant improvement in overall survival for patients receiving tislelizumab plus chemotherapy compared with those receiving chemotherapy alone (median OS, 15.0 versus 12.9 months; HR, 0.80 [95% CI, 0.70–0.92]; *p* = 0.001) [7]. Therefore, first-line therapy for metastatic GECs should incorporate immune checkpoint inhibitors (ICIs) alongside standard-of-care chemotherapy in patients without contraindications whose tumors are PD-L1 positive or exhibit microsatellite instability (MSI) [8]. Different PD-L1 scoring systems are used to determine the positivity rate. These include Tumor Proportion Score (TPS), corresponding to the percentage of viable tumor cells that show partial or complete membrane staining for PD-L1 at any intensity, and the Combined Positive Score, known as the ratio of PD-L1-expressing cells (including tumor cells, lymphocytes, and macrophages) to the total number of viable tumor cells [9]. At a cutoff of ≥1, the CPS metric identified 57.6% of patients as PD-L1-positive, compared with only 12.5% with TPS. Ultimately, an objective response rate (ORR) of 15.5% [95% CI, 10.1–22.2%] within the CPS ≥ 1 cohort served as the basis for the FDA accelerated approval of pembrolizumab in this third-line setting [10,11].

However, determining true PD-L1 status remains challenging because endoscopic biopsy specimens may not accurately represent the entire tumor. Small tissue fragments obtained during endoscopy frequently lead to misclassification of a patient’s actual PD-L1 status. An analysis of 112 gastric cancer samples demonstrated a concordant classification rate of only 63.4% between preoperative biopsies and non-treated matched surgical resection specimens [12,13]. Tumors demonstrate dynamic changes in biomarker expression and variable histological features. These phenomena are referred to as temporal and spatial heterogeneity, which can be further classified as intratumoral heterogeneity, occurring within a single tumor, and as primary and metastatic heterogeneity, observed between primary and metastatic sites [14]. Paired analyses of primary gastric tumors and corresponding metastases demonstrate marked spatial heterogeneity, resulting in a PD-L1 concordance rate of only 61% (38/62) [15]. As a result, a small biopsy taken from just one area of the primary tumor is likely to either underestimate or overestimate its actual biomarker status, especially when the decision-making thresholds such as CPS ≥ 1 or ≥5 are involved. Another difficulty arises from temporal discordance in PD-L1 expression, likely due to inflammatory cytokines produced by infiltrating immune cells and to chemotherapy-induced remodeling of the tumor microenvironment [16]. Indeed, post-chemotherapy evaluations demonstrated a discordance rate reaching 50.0% (36/72 patients) [16]. Further prospective studies remain essential to fully quantify these discordant rates and clarify their impact on predicting response to systemic therapy.

Rationale for the review: Although immune checkpoint inhibitors have been successfully integrated into gastroesophageal cancer treatment, baseline PD-L1 evaluation remains clinically challenging. PD-L1 expression demonstrates substantial spatial and temporal heterogeneity, and small endoscopic biopsies frequently fail to reflect the true status, resulting in notable discordance with surgical resections. Additionally, systemic therapy can induce significant post-treatment changes in PD-L1 expression, yet clinical decisions often continue to rely on archival tissue. Therefore, a systematic evaluation of treatment-induced PD-L1 dynamics in both neoadjuvant and metastatic settings is necessary to prevent treatment selection based on outdated biomarker profiles and to optimize immunotherapy strategies.

Aim of the review: This review aims to synthesize current clinical evidence on treatment-induced shifts in PD-L1 expression in gastroesophageal cancer and examine the literature to elucidate the underlying biological and clinical mechanisms driving these dynamic alterations.

## 2. Materials and Methods

### 2.1. Search Strategy

**Figure 1:**
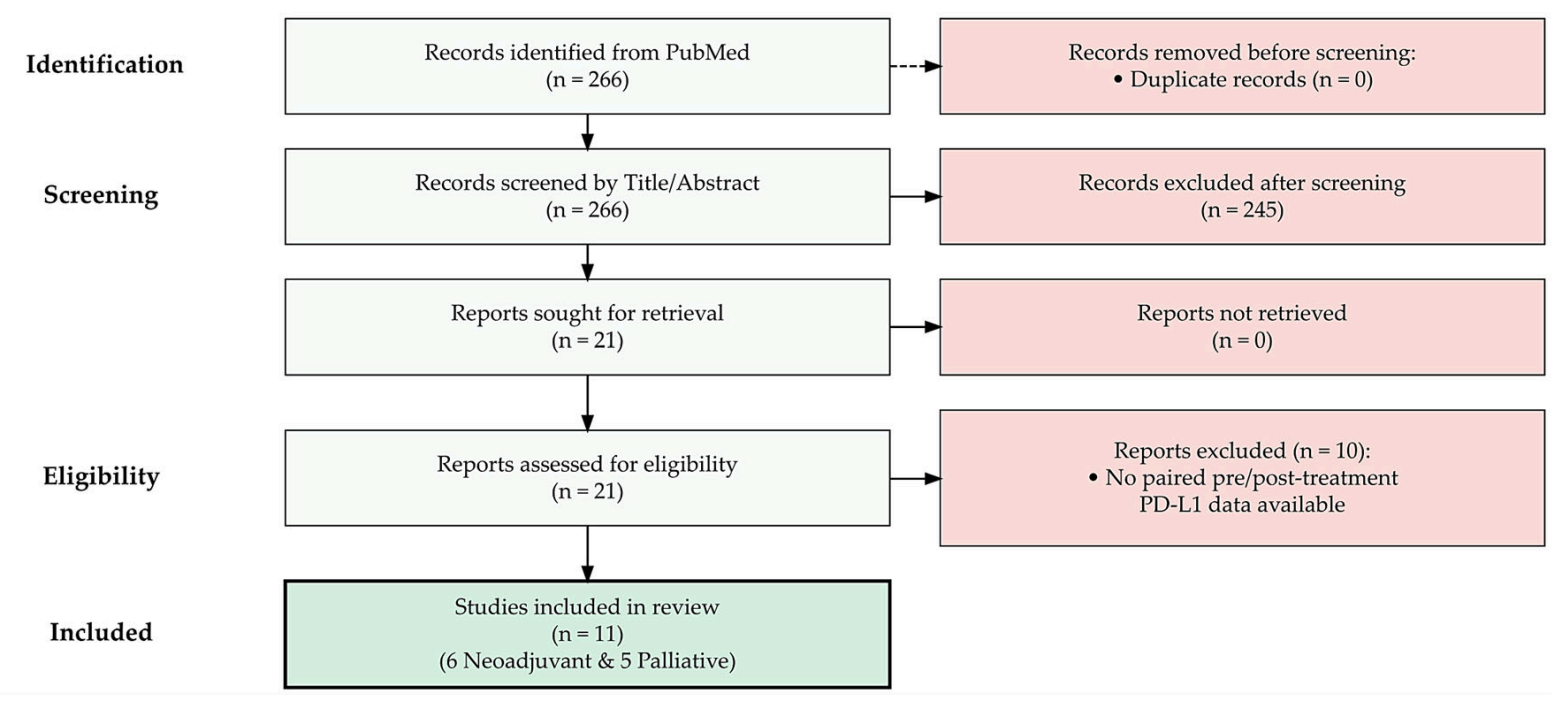
PRISMA 2020 flow diagram demonstrating the literature search, screening, and selection process.

This study was conducted and reported in accordance with the PRISMA 2020 guidance (Table S1). A comprehensive literature search was conducted in the electronic database PubMed/MEDLINE to identify studies published up to May 2026 (Figure 1). The search strategy included combinations of the following Medical Subject Headings (MeSH) and free-text keywords: (PD-L1 OR “programmed death ligand 1”) AND (heterogeneity OR dynamic OR temporal OR spatial) AND (gastric cancer OR gastroesophageal OR esophageal). Two reviewers independently screened all retrieved titles, abstracts, and full-text articles against the pre-defined inclusion and exclusion criteria. Disagreements between reviewers were resolved by consultation with a third senior reviewer.

### 2.2. Eligibility Criteria

Studies were included if they met the following criteria: investigated patient cohorts with histologically confirmed gastroesophageal or gastric cancer; evaluated PD-L1 expression in GC; provided paired data or comparative pre- and post-treatment PD-L1 status. Review articles, case reports, animal studies, conference abstracts without full-text availability, and non-English language articles were excluded.

### 2.3. Data Extraction

Data extraction was completed using a primary extractor and an independent verifier for all eligible studies. The extracted parameters included: study and patient characteristics (author, year of publication, study design, sample size, median age, sex, and tumor location); clinicopathological data (tumor stage, histological type, differentiation grade, and baseline biomarker status); treatment detail (regimen and line of therapy); PD-L1 dynamics (concordance in PD-L1 expression, including its appearance or loss after treatment); clinical outcomes (objective response rate (ORR), disease-free survival (DFS)).

### 2.4. Statistical Analysis

Due to the heterogeneity in study designs and PD-L1 assessment methods, a formal meta-analysis was not performed. The narrative synthesis and qualitative data transformation were structured in accordance with the Synthesis Without Meta-analysis (SWiM) reporting guidelines and PRISMA 2020 standards. Proportion data, including pre-treatment/post-treatment PD-L1 positivity rates and paired dynamic alterations (PD-L1 gain and PD-L1 loss), were expressed as percentages with corresponding exact 95% confidence intervals (95% CIs) calculated using the Clopper-Pearson method. Statistical significance of paired changes was assessed using McNemar’s exact test. All analyses and graphical representations were performed using RStudio (version 2026.01.0).

### 2.5. Protocol Registration and Risk-of-Bias Assessment

This systematic review was retrospectively registered on the Open Science Framework (OSF): https://osf.io/b6u83 (accessed on 29 August 2026). Study-level risk of bias was assessed using the Newcastle-Ottawa Scale, with the full appraisal provided in Table S2.

## 3. Results

### 3.1. Study Characteristics

The systematic search following PRISMA guidelines revealed 266 articles in the initial electronic search; 245 were excluded after screening the article titles and abstracts. A total of 21 potentially eligible studies were selected for detailed evaluation, 10 of which were excluded due to insufficient reported data (Figure 1). Finally, 11 studies from 2018 to 2026 were included in the review (Table 1): three of them are prospective phase II trials (Kelly et al. [17]; Li et al. [18]; Lim et al. [19]), one is a prospective phase I trial (Chong et al. [20]), while the other ones are retrospective.

The methodological quality of the 11 included studies was assessed using the adapted Newcastle-Ottawa Scale (Table S2). Overall, 10 studies were categorized as low risk of bias, while 1 study was categorized as moderate risk.

### 3.2. Neoadjuvant Setting for Locally Advanced Disease

We identified six distinct clinical cohorts analyzing neoadjuvant interventions in locally advanced gastroesophageal cancers (Table 2). The total sample size across these studies was 654 patients, with sample sizes ranging from 25 patients (Li et al. [18]) to 173 patients (Soeratram et al. [23]), and a total of 430 patients with paired biopsies. Data regarding Stage III disease were presented in three studies, reaching the maximum in Li et al. [18] (100%; 25/25) and Ribeiro et al. [22] (96.77%; 150/155), while Kelly et al. [17] reported only 37.5% (12/32) of current stage. In terms of therapeutic protocols, two investigations (Kelly et al. [17], Soeratram et al. [23]) utilized combination strategies consisting of neoadjuvant chemotherapy plus chemoradiotherapy, while four others used systemic therapy only. Three studies reported utilizing anti-PD-1 treatment (Kelly et al. [17], Li et al. [18], Yu et al. [24]). All studies used immunohistochemistry (IHC) to evaluate PD-L1 expression in biopsy and surgical/resected tissue samples, while PD-L1 antibody clones varied across cohorts. Five studies reported a CPS ≥ 1 cut-off, while Zhao et al. [21] had a CPS ≥ 5 cut-off.

### 3.3. Metastatic Disease

For the advanced and metastatic setting, 5 independent studies were included, with a total population of 371 patients (Table 3). The sample sizes ranged between 16 patients (Lim et al. [19]) and 189 patients (Zhou et al. [15]). As presented in Table 2, four included cohorts evaluated different systemic regimens, relying on first-line chemotherapy, and only Chong et al. [20] evaluated both first- and second-line chemotherapies. Three studies (Chong et al. [20], Lim et al. [19], Massa et al. [25]) reported the use of anti-PD-1 treatment regimens. Four studies used immunohistochemistry (IHC) to evaluate PD-L1 expression in biopsy tissue samples. In contrast, one study (Chong et al. [20]) used subtraction enrichment (SE)-iFISH analysis to evaluate PD-L1 expression on circulating tumor cells (CTC) in peripheral blood samples. Three studies (Lim et al. [19]), Massa et al. [25], Zhou et al. [15]) reported using a CPS ≥ 1 cut-off, while Chong et al. [20] used a positivity threshold defined as ≥10% PD-L1+ CTCs in SE-iFISH analysis.

### 3.4. Neoadjuvant Efficacy and Pathological Response

Pathological efficacy and survival outcomes for the neoadjuvant interventions are detailed in Table 4. Among studies reporting pathological complete response (pCR) rates, the highest rate was reported by Kelly et al. [17], with pCR in 31.03% (9/29 reported). In this study, patients receiving just Arm A had a superior pCR rate (6/15) than those in Arm B (3/14). In contrast, Zhao et al. [21] reported a pCR rate of 3.84% (4/104) with 3-cycle oxaliplatin and tegafur/gimeracil/oteracil NACT. Li et al. [18] documented an intermediate pCR rate of 15.78% (3/18), Yu et al. [24] presented 17.9% (29/162), and Ribeiro et al. [22] reported 9.7% (15/74). The median follow-up period ranged from 24.7 (Li et al. [18]) to 60.3 months (Ribeiro et al. [22]). Long-term survival outcomes were reported in only two studies; therefore, a comparison among studies is not possible.

### 3.5. Efficacy in the Metastatic Setting and Objective Responses

Therapeutic response rates and survival outcomes for metastatic disease are detailed in Table 5. Objective response rates (ORR) were disclosed in two key studies. Lim et al. [19] reported an ORR of 68.75% in first-line treatment, while Yang et al. [16] registered an ORR of 48.61%. Partial Response (PR) was observed in 44.44% (32/72) of patients in Yang et al. [16], 59.72% (43/72) in Chong et al. [20], and 68.75% (11/16) in Lim et al. [19]. Progressive Disease (PD) was recorded by Yang et al. [16] at 20.83% (15/72), followed by Chong et al. [20] at 15.27% (11/72), and Lim et al. [19] at 12.5% (2/16). Survival endpoints in the current cohort showed a median progression-free survival (PFS) of 11.9 months (5.12–18.28 months) in Lim et al. [19], compared with 7.03 months in Chong et al. [20].

### 3.6. Alterations in PD-L1 Positivity Rates (Pre- vs. Post-Treatment)

All 11 included studies contributed to the synthesis. Data from paired pre- and post-treatment samples were used to compare the PD-L1 positivity rate across studies. Because the included studies applied different CPS thresholds (≥1, ≥5, and ≥10), and one study (Chong et al. [20]) assessed PD-L1 on circulating tumor cells by SE-iFISH rather than on tissue by immunohistochemistry, the pooled positivity shifts reported below should be read as directional signals rather than quantitatively comparable rates.

**Figure 2:**
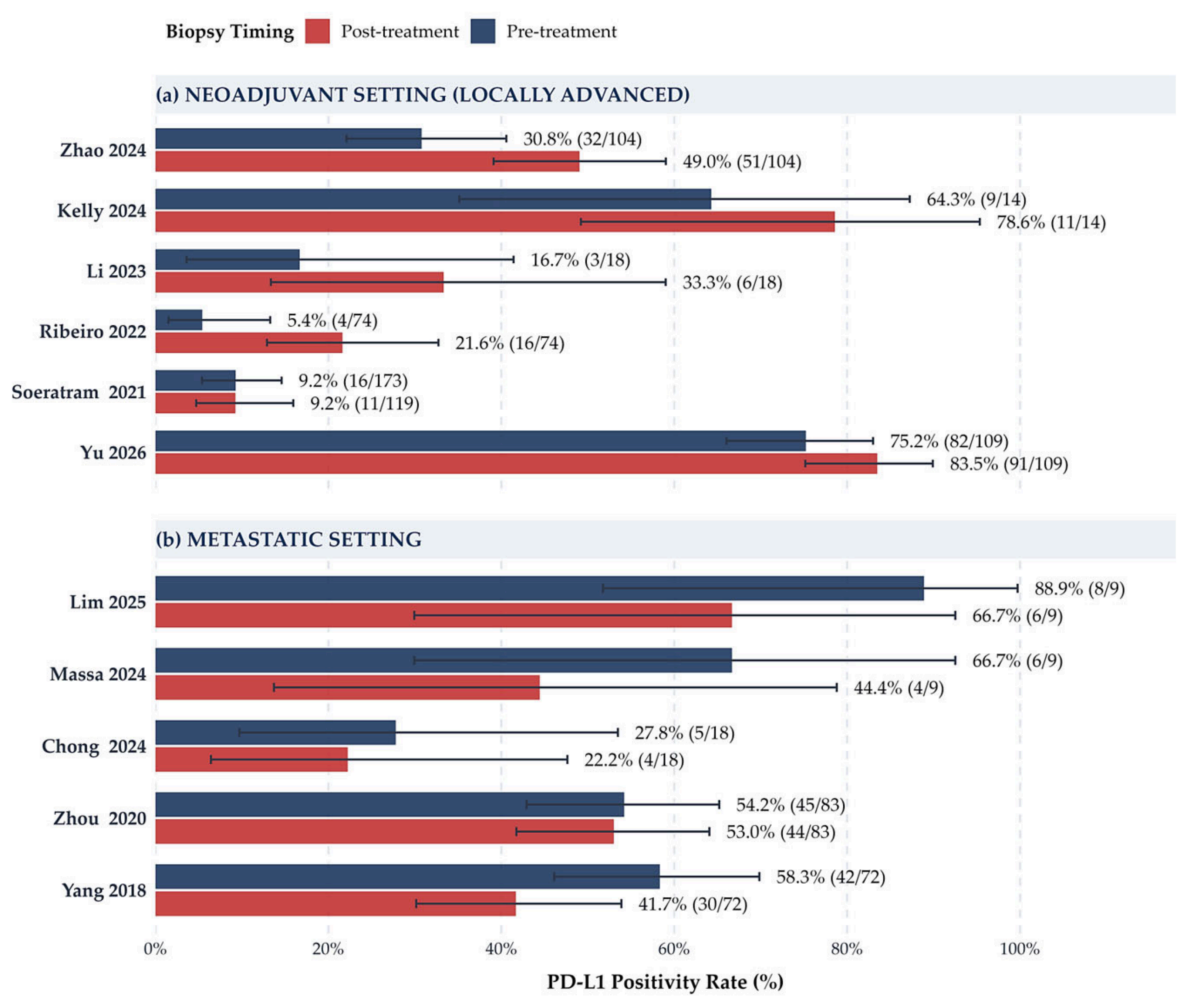
Grouped bar chart with 95% confidence intervals representing PD-L1 positivity rates before and after treatment across included studies. Rates are presented separately for: (**a**) neoadjuvant treatment setting (locally advanced cases); (**b**) metastatic setting. Absolute patient numbers (*n*/*N*) and exact percentages are provided adjacent to each bar. Data are presented from Zhao et al. [21], Kelly et al. [17], Li et al. [18], Ribeiro et al. [22], Soeratram et al. [23], Yu et al. [24], Lim et al. [19], Massa et al. [25], Chong et al. [20], Zhou et al. [15], Yang et al. [16].

The proportion of PD-L1–positive cases before and after systemic therapy is illustrated in Figure 2. In the neoadjuvant setting (locally advanced disease), a clear and consistent trend toward increased PD-L1 positivity post-treatment was observed across nearly all cohorts. Specifically, Zhao et al. [21] demonstrated a substantial rise in positivity from 30.8% [95% CI: 22.1–40.6%] (32/104) pretreatment to 49.0% [95% CI: 39.1–59.0%] (51/104) post-treatment. Similarly, Ribeiro et al. [22] reported a nearly fourfold increase, rising from 5.4% [95% CI: 1.5–13.3%] (4/74) to 21.6% [95% CI: 12.9–32.7%] (16/74). Upward shifts were also confirmed by Kelly et al. [17] (from 64.3% [95% CI: 35.1–87.2%] to 78.6% [95% CI: 49.2–95.3%]), Li et al. [18] (from 16.7% [95% CI: 3.6–41.4%] to 33.3% [95% CI: 13.3–59.0%]), and Yu et al. [24] (from 75.2% [95% CI: 66.0–83.0%] to 83.5% [95% CI: 75.2–89.9%]). The only exception in this setting was Soeratram et al. [23], which did not report numbers of paired pre- and post-treatment PD-L1 positivity, and non-paired analysis of PD-L1 expression remained stable (9.2% [95% CI: 5.4–14.6%] pre- vs. 9.2% [95% CI: 4.7–15.9%] post-treatment).

Conversely, in the metastatic setting, chemotherapy and systemic intervention were associated with a downward or stable trajectory of PD-L1 positivity in all five evaluated studies. Lim et al. [19] reported a reduction from 88.9% [95% CI: 51.8–99.7%] (8/9) to 66.7% [95% CI: 29.9–92.5%] (6/9), while Massa et al. [25] observed a decline from 66.7% [95% CI: 29.9–92.5%] (6/9) to 44.4% [95% CI: 13.7–78.8%] (4/9). Yang et al. [16] documented a drop from 58.3% [95% CI: 46.1–69.8%] (42/72) to 41.7% [95% CI: 30.2–53.9%] (30/72), and Chong et al. [20] registered a shift from 27.8% [95% CI: 9.7–53.5%] (5/18) to 22.2% [95% CI: 6.4–47.6%] (4/18). Finally, Zhou et al. [15] demonstrated stable positivity rates before and after therapy (54.2% [95% CI: 42.9–65.2%] vs. 53.0% [95% CI: 41.7–64.1%], respectively).

### 3.7. Characterization of Dynamic Immunophenotypic Shifts

**Figure 3:**
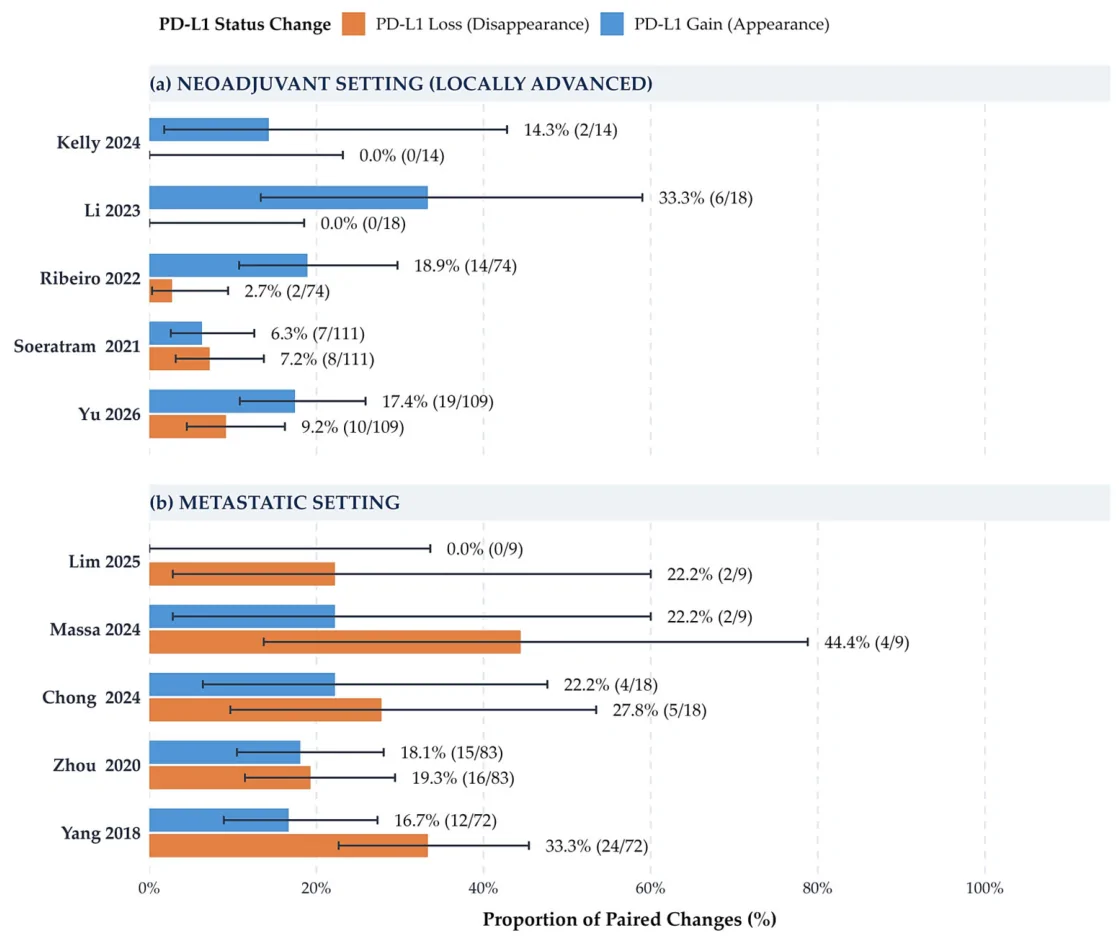
Grouped bar chart with 95% confidence intervals (CI) showing treatment-induced changes in PD-L1 expression dynamics across paired biopsies. Proportions of PD-L1 gain (appearance) and loss (disappearance) are displayed separately for: (**a**) neoadjuvant treatment setting (locally advanced); (**b**) metastatic setting. Absolute patient numbers (*n*/*N*) and exact percentage rates are indicated adjacent to each bar. Data are presented from Kelly et al. [17], Li et al. [18], Ribeiro et al. [22], Soeratram et al. [23], Yu et al. [24], Lim et al. [19], Massa et al. [25], Chong et al. [20], Zhou et al. [15], Yang et al. [16].

We evaluated the proportion of tumors that experienced either an absolute increase in PD-L1 expression (negative-to-positive conversion) or a complete loss of baseline positivity (positive-to-negative conversion) after systemic therapy (Figure 3). Paired biopsy data were available for 10 of the 11 included studies (Zhao et al. [21] was not included because it did not report immunophenotypic shifts).

In the neoadjuvant setting a prominent trend toward PD-L1 appearance was observed across all cohorts, with the highest conversion rate reported by Li et al. [18] (33.3%, 6/18; 95% CI: 13.3–59.0%), followed by Ribeiro et al. [22] (18.9%, 14/74; 95% CI: 10.7–29.7%), Yu et al. [24] (17.4%, 19/109; 95% CI: 10.8–25.9%), Kelly et al. [17] (14.3%, 2/14; 95% CI: 1.8–42.8%), and Soeratram et al. [23] (6.3%, 7/111; 95% CI: 2.6–12.6%). Notably, in small cohorts such as Kelly et al. [17] and Li et al. [18], no patients experienced loss of baseline PD-L1 expression (0.0%), likely due to limited sample size. Conversely, larger cohorts such as Yu et al. [24], Soeratram et al. [23], and Ribeiro et al. [22] displayed a dual phenomenon where a subset of patients lost baseline positivity (9.2% [95% CI: 4.5–16.2%], 7.2% [95% CI: 3.2–13.7%], and 2.7% [95% CI: 0.3–9.4%], respectively), reflecting marked intratumoral heterogeneity and divergent treatment-induced selective pressures.

In contrast, the metastatic setting demonstrated a marked shift toward PD-L1 loss (Figure 3). The rate of PD-L1 loss was particularly pronounced in studies by Massa et al. [25] (44.4%, 4/9; 95% CI: 13.7–78.8% vs. 22.2% gain) and Yang et al. [16] (33.3%, 24/72; 95% CI: 22.7–45.4% vs. 16.7% gain). Lim et al. [19] exhibited an isolated pattern of PD-L1 loss (22.2%, 2/9; 95% CI: 2.8–60.0%) with a complete absence of new PD-L1 gain (0.0%). Meanwhile, Chong et al. [20] and Zhou et al. [15] demonstrated comparable rates of biomarker gain and loss (22.2% vs. 27.8% and 18.1% vs. 19.3%, respectively).

Detailed numeric data including exact sample sizes, event counts, percentages, and corresponding Clopper-Pearson 95% confidence intervals for all cohorts are provided in Tables S3 and S4.

## 4. Discussion

The findings indicate that treatment alters PD-L1 expression in gastroesophageal cancer; however, this effect is highly dependent on the clinical context. Opposite trends in biomarker modulation were observed between the neoadjuvant and metastatic cohorts, underscoring the importance of dynamic monitoring of PD-L1 status throughout treatment. It is important to note that the treatment protocols in the various studies were diverse and included chemotherapy, chemoradiotherapy, immune checkpoint inhibitors, and anti-HER2 therapies. As a result, the observed changes in PD-L1 expression may be due not only to different disease settings (neoadjuvant vs. metastatic) but also to specific biological mechanisms induced by treatment, such as radiation-induced immunogenic cell death or ICI-mediated selective pressure. Notably, in an included study Chong et al. captured similar heterogeneous shifts using SE-iFISH to assess PD-L1 on circulating tumor cells (CTCs), demonstrating that non-invasive liquid profiling reflects the same temporal heterogeneity [20].

### 4.1. Interpretation of Findings in Neoadjuvant Cohort

In the neoadjuvant cohort, we observed an upward trend in PD-L1 expression following treatment. Several factors may account for these changes. First, it is important to note that the primary tumor sample was obtained via endoscopic biopsy, while the subsequent sample was derived from the entire resection specimen. Although the European Society of Gastrointestinal Endoscopy recommends obtaining at least six biopsies from tumor tissue, these are typically collected from peripheral regions rather than the tumor center. In contrast, the resected specimen after gastrectomy allows analysis of central and inaccessible tumor regions for endoscopic biopsy, with manual selection of the most representative one or two central tissue blocks for PD-L1 scoring [26,27]. The spatial heterogeneity of gastric cancer warrants consideration. A Japanese study retrospectively re-evaluated PD-L1 expression, comparing biopsy and resected specimens in patients who did not receive neoadjuvant therapy. The findings were inconclusive, with PD-L1 positivity observed in 89 of 191 biopsy samples (46.6%) and 135 of 191 resected samples (70.1%), resulting in a concordance rate of 64.4% [13]. These findings are consistent with our comprehensive analysis and underscore the diagnostic challenges associated with spatial biomarker variability. Nevertheless, definitive conclusions necessitate larger prospective comparisons between pretreated and untreated cohorts. To address sampling errors, an additional analysis of 152 gastric cancer specimens found that obtaining at least five biopsy samples significantly reduced discordance, resulting in a concordance rate of 92.4% [95% CI, 87.8–97.0%] with surgical specimens, compared to 85.7% [95% CI, 79.8–91.6%] when only four samples were collected [28]. Additionally, the use of different assays for PD-L1 assessment, such as 22C3 and 28-8, may account for the observed changes.

**Figure 4:**
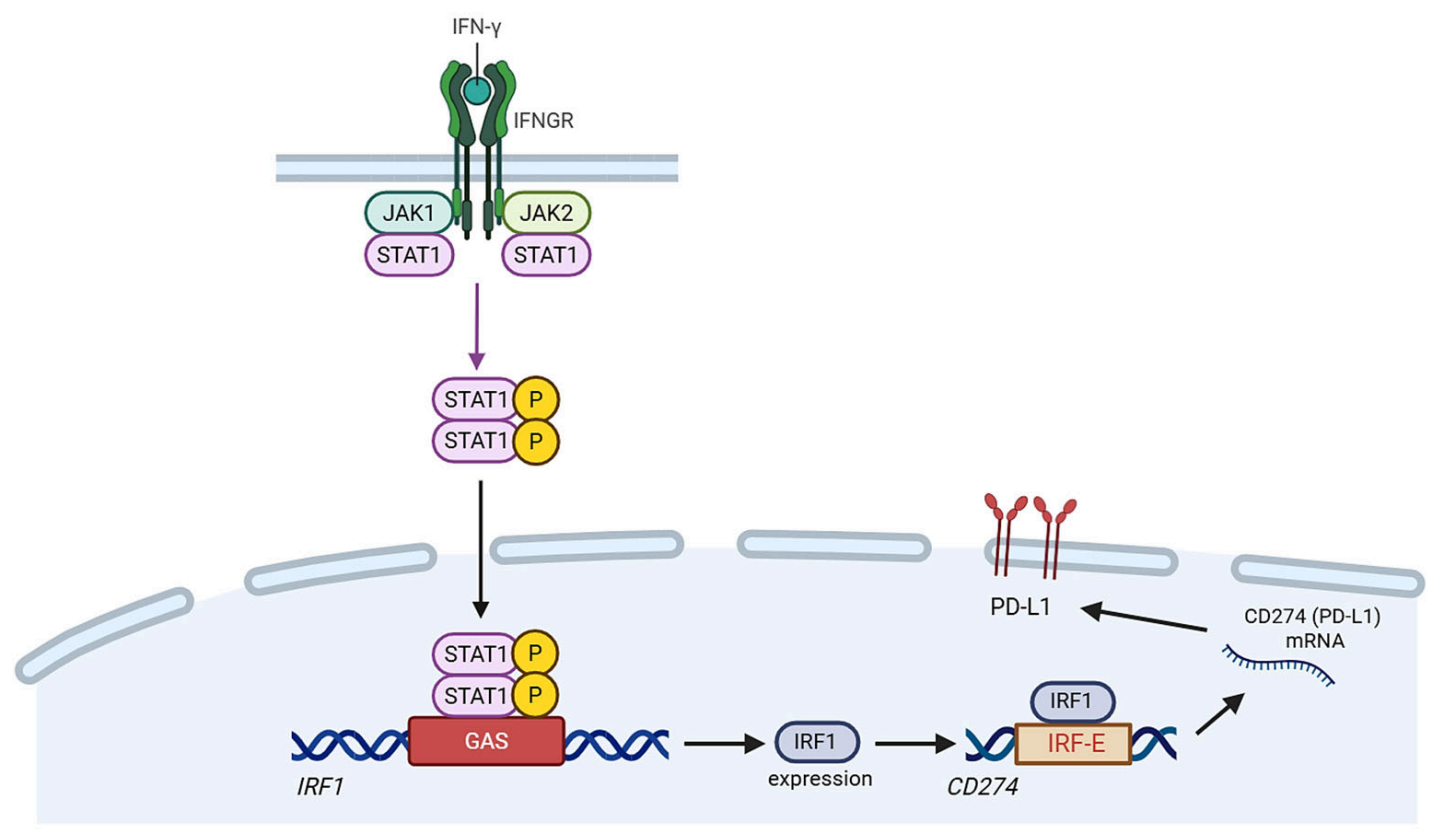
Mechanism of IFN-γ/JAK–STAT1 pathway-mediated CD274 (PD-L1) expression in gastric cancer. Binding of extracellular IFN-γ to the IFNGR complex triggers phosphorylation of JAK1 and JAK2, which recruit and activate STAT1. Phosphorylated STAT1 homodimers translocate to the nucleus and bind the GAS element to initiate IRF1 transcription. The synthesized IRF1 protein then binds to the IRF-E site on the CD274 promoter, driving CD274 mRNA production and subsequent surface transport of PD-L1, thereby establishing adaptive immune resistance. Black arrows indicate molecular activation, translocation, or transition steps, while yellow circles with ‘P’ represent protein phosphorylation. Abbreviations: CD274—cluster of differentiation 274; GAS—gamma-interferon activated sequence; IFN-γ—interferon-gamma; IFNGR—interferon-gamma receptor; IRF-E—interferon regulatory element; IRF1—interferon regulatory factor 1; JAK1—Janus kinase 1; JAK2—Janus kinase 2; mRNA—messenger ribonucleic acid; PD-L1—programmed cell death ligand 1; STAT1—signal transducer and activator of transcription 1. Created in BioRender. Khaustova, M. (2026) https://BioRender.com/ jdoxz1f.

A further explanation for PD-L1 upregulation may involve biological changes induced by chemotherapy. Neoadjuvant treatment is recognized for causing immunogenic cell death (ICD), which elicits an immune response against antigens from dead cells [29]. These antigens subsequently promote extensive polyclonal T-cell activation, including both CD8+ (cytotoxic) and CD4+ (helper) subsets, and infiltration into the tumor microenvironment, leading to IFN-γ release [27,28,29]. The IFN-γ/JAK–STAT1 pathway then stimulates transcription of CD274 (PD-L1) mRNA, resulting in overexpression of the PD-L1 protein (Figure 4) [30,31]. A study of 60 participants found significant increases in median expression levels of PD-1 (0.68% vs. 5.19%, *p* < 0.001), PD-L1 (0.26% vs. 1.06%, *p* = 0.008), CD4 (5.50% vs. 21.38%, *p* < 0.001), CD8 (2.20% vs. 12.09%, *p* < 0.001), and TIM3 (0.24% vs. 2.47%, *p* < 0.001) after neoadjuvant chemotherapy. These findings may support a positive correlation between tumor immune cell infiltration and checkpoint molecule expression [32]. Another study (*n* = 30) also demonstrated increased numbers of CD3+ T cells, CD8+ T cells, and NK cells in the tumor stroma after neoadjuvant chemoimmunotherapy in patients exhibiting a major pathological response [33]. However, further prospective studies with comprehensive subgroup analyses are required to validate these findings and clarify any correlations.

Additionally, PD-L1 is expressed not only by tumor cells, but also by macrophages, dendritic cells, and other immune or stromal cells. This heterogeneity complicates the measurement of tumor-specific PD-L1 expression and the assessment of its precise clinical relevance [34]. Beyond gene transcription, PD-L1 protein levels are regulated by translation, post-translational modifications such as glycosylation, phosphorylation, and ubiquitination, and cellular degradation. Major signaling pathways involved in this regulation include EGFR–PI3K–AKT–mTOR, MAPK, JAK–STAT, NF-κB, and GSK3β, along with ubiquitin ligases and deubiquitinases [35,36]. Chemotherapy can induce DNA damage, activate stress signaling, and promote cytokine release, all of which may directly increase PD-L1 protein expression.

### 4.2. Interpretation of Findings in Metastatic Cohort

Conversely, in the metastatic setting, a trend toward PD-L1 downregulation was observed. PD-L1 loss was consistently reported across all metastatic studies, with rates ranging from 19.3% to 44.4%. These findings indicate that loss of PD-L1 expression is a consistent feature in the metastatic setting for the studies analyzed. For this setting, the concept of spatial intratumoral heterogeneity should be expanded to include intertumoral heterogeneity, which refers to biomarker differences between the primary tumor site and metastatic sites. These differences are challenging to capture using biopsy specimens alone [37].

**Figure 5:**
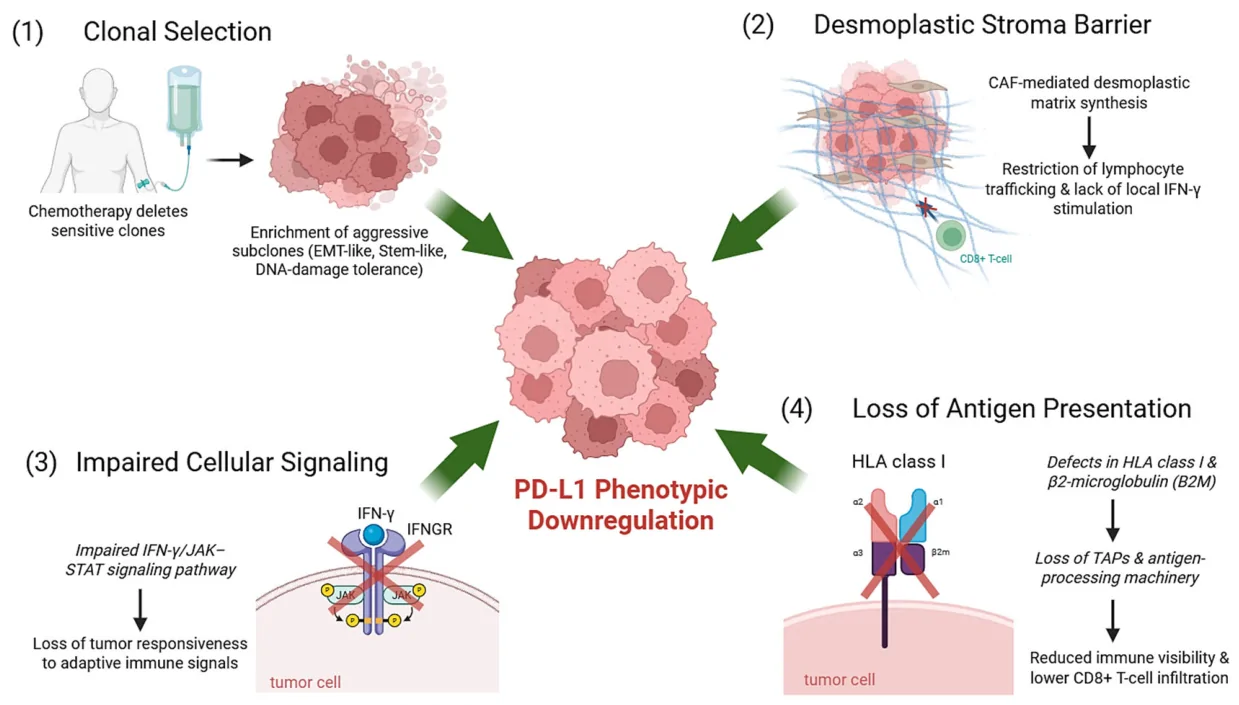
Proposed multifactorial drivers of PD-L1 downregulation in metastatic gastric cancer. Central PD-L1 loss may be driven by: (1) Darwinian clonal selection where chemotherapy eradicates sensitive cells, enriching aggressive EMT- and stem-like clones; (2) desmoplastic stroma synthesized by cancer-associated fibroblasts (CAFs), which restricts CD8+ T-cell trafficking; (3) impairment of the IFN-γ/JAK–STAT signaling cascade, preventing adaptive PD-L1 upregulation; and (4) loss of the antigen-presentation machinery (HLA class I, B2M, TAPs), reducing immune visibility and lymphocyte infiltration. Thin black arrows indicate cause-and-effect steps, while large green arrows point to the central outcome. Abbreviations: B2M—beta-2 microglobulin; CAFs—cancer-associated fibroblasts; EMT—epithelial–mesenchymal transition; HLA—human leukocyte antigen; IFN-γ—interferon-gamma; JAK–STAT—Janus kinase–signal transducer and activator of transcription; PD-L1—programmed cell death ligand 1; TAPs—transporters associated with antigen processing. Created in BioRender. Khaustova, M. (2026) https://BioRender.com/ mffgyby.

Several interconnected biological processes may contribute to PD-L1 temporal changes (Figure 5). According to the concept of Darwinian clonal selection, chemotherapy can eliminate all sensitive clones. Concurrently, subclones exhibiting increased DNA-damage tolerance, modified apoptotic pathways, Epithelial–Mesenchymal Transition (EMT)-like characteristics, stem-like features, or enhanced stromal support may persist and subsequently display more aggressive behavior. Furthermore, the surviving cell population may demonstrate an altered interferon response, resulting in a distinct PD-L1 expression profile. Chemotherapy does not need to directly repress PD-L1 in every surviving cell; rather, it may alter the overall tumor composition [38,39]. However, limited clinical outcome data were available for analysis to establish correlations between PD-L1 loss and disease prognosis.

Organotropic metastatic heterogeneity, driven by site-specific interactions between cancer cells and the local microenvironment, may also contribute to PD-L1 phenotypic downregulation [40]. Peritoneal, liver, lung, and nodal lesions exhibit distinct cytokine profiles, extracellular matrices, oxygenation, nutrient availability, resident macrophages, fibroblasts, and vascular barriers. These localized features determine whether T cells can infiltrate the lesion and whether tumor cells receive interferon-gamma (IFN-γ) signaling, the primary adaptive driver of PD-L1 induction [41]. Additionally, cancer-associated fibroblasts (CAFs) within the metastatic niche synthesize a dense desmoplastic stroma composed of collagen, fibronectin, proteoglycans, and hyaluronic acid. This extracellular matrix forms a physical and biochemical barrier that restricts lymphocyte trafficking [42]. In addition, metastatic clones may reduce immune visibility by losing components of the antigen-presentation pathway: human leukocyte antigen (HLA) class I, β2-microglobulin (B2M), transporters associated with antigen processing (TAPs), antigen-processing machinery, or IFN-γ/JAK–STAT signaling [43]. This molecular loss reduces sensitivity to IFN-γ stimulation, thereby lowering PD-L1 expression. Supporting this mechanism, negative expression of HLA-A/B/C and β2M in gastric tumors was associated with reduced CD8+ T cell infiltration, EBV-negativity, decreased PD-L1 expression (*p* < 0.001), and poorer survival outcomes (*p* = 0.019) [44].

### 4.3. Statistical Interpretation and Methodological Considerations

Interpretation of PD-L1 dynamics requires careful consideration of sample size and statistical power. Studies with small cohorts (*n* < 20, e.g., Kelly et al., Li et al., Massa et al., and Lim et al. [17,18,19,25]) frequently report wide and overlapping 95% confidence intervals. Although overlapping confidence intervals in paired data do not categorically exclude statistical significance (*p* < 0.05), the substantial uncertainty renders these studies underpowered individually. However, clear trends appear when looking at larger studies and synthesized data. In the neoadjuvant setting, larger cohorts (e.g., Ribeiro et al., *n* = 74; Zhao et al., *n* = 104 [21,22]) demonstrate significant PD-L1 upregulation, with gains consistently surpassing losses. This consistent pattern reflects a primary biological effect, likely attributable to spatial heterogeneity and a treatment-induced inflammatory response, rather than random sampling variation. Conversely, metastatic cohorts (e.g., Zhou et al., Chong et al., and Yang et al. [15,16,20]) exhibit balanced rates of PD-L1 gain and loss. The lack of a consistent directional change in these cohorts reflects pronounced spatial-temporal heterogeneity and ongoing clonal evolution during systemic therapy.

### 4.4. Clinical Implications

Our results highlight the clinical utility of longitudinal PD-L1 assessment in patients with metastatic disease. Relying on a single baseline biopsy may lead to an inaccurate assessment of immunotherapy eligibility, particularly when phenotypic shifts occur mid-treatment. Identifying these biomarker dynamics suggests that serial evaluation could refine patient stratification and improve therapeutic outcomes.

### 4.5. Limitations and Future Directions

This study has several limitations. First, several of the evaluated studies suffer from relatively small sample sizes of paired biopsies, which limit the statistical power of the observed percentages. Second, substantial methodological heterogeneity was observed, particularly in therapeutic regimens and inconsistently reported biomarker data. Moreover, primary discrepancies in PD-L1 status may be related to technical variations in its assessment, which were outside the analytical scope of this study. These include the use of different types of diagnostic antibodies (e.g., 22C3, SP263) and distinct scoring methodologies. Additionally, publication bias cannot be entirely excluded, as studies with negative findings on PD-L1 stability may be underrepresented in the published literature. Finally, this systematic review was registered retrospectively, but not prospectively, on the Open Science Framework (OSF) (https://osf.io/b6u83; accessed on 29 August 2026), which should be considered when interpreting its findings.

To address these limitations, future research should prioritize standardized, prospective evaluations of longitudinal PD-L1 dynamics. In particular, non-invasive approaches such as liquid biopsy (e.g., circulating tumor cells or cell-free nucleic acids) offer a promising avenue to overcome the clinical constraints of repeated tissue sampling.

## 5. Conclusions

This systematic review confirms that PD-L1 expression in gastroesophageal cancer is highly dynamic, demonstrating context-dependent shifts following systemic therapy. While neoadjuvant treatment consistently upregulates PD-L1 expression by inducing an inflamed tumor state, in the metastatic setting the direction of change was more heterogeneous but consistent with a marked predominance of PD-L1 loss. These trends provide a clear rationale for longitudinal biomarker monitoring via tissue re-biopsies or liquid biopsies, as it is essential to prevent targeting obsolete tumor phenotypes.

## Figures and Tables

**Table 1:** Baseline Clinical and Demographic Characteristics of Patients of All Included Studies.

Study (Author, Year)	Country	Design	Sample Size (*N*)	Patients with Paired Biopsies (*n*)	Tumor Location	Setting Type
Zhao 2024 [21]	China	retrospective	104	104	GC, locally advanced	NACT
Kelly 2024 [17]	USA	prospective trial phase II	32	14	EC/GEJC	NACT + CR
Li 2023 [18]	China	prospective trial phase II	25	18	GC, locally advanced	NACT
Ribeiro 2022 [22]	Brazil	retrospective	155	74	GEC, locally advanced	NACT
Soeratram 2021 [23]	The Netherlands	retrospective	173	111	EC	NACT + CR
Yu 2026 [24]	China	retrospective	165	109	GC/GEJC	NACT
Lim 2025 [19]	Republic of Korea	prospective trial phase II	16	9	GC, advanced	Metastatic (first-line)
Massa 2024 [25]	Italy	retrospective	22	9	GEC, advanced	Metastatic (first-line)
Chong 2024 [20]	China	prospective trial phase I	72	18	GC, advanced	Metastatic (first or second line)
Zhou 2020 [15]	USA	retrospective	189	83	GEC (locally advanced and advanced)	Metastatic (first-line)
Yang 2018 [16]	Republic of Korea	retrospective	72	72	GC, advanced	Metastatic (first-line)

EC—esophageal cancer; GEJC—gastroesophageal junction cancer; GC—gastric cancer; GEC—gastroesophageal cancer; NACT—neoadjuvant chemotherapy; CR—chemoradiation.

**Table 2:** Baseline Clinical and Demographic Characteristics of Patients in the Neoadjuvant Setting.

Study (Author, Year)	Sample Size (*N*)	Age (Median/Range)	Stage III, *n* (%)	Setting Type	Therapeutic Regimen/Interventions	Anti PD-1 Treatment	Biomarker Assay; CPS Cut-Off;	PD-L1 Antibody Clone
Zhao 2024 [21]	104	54.48 ± 12.4 (mean age + SD)	NA	NACT	Oxaliplatin and tegafur/gimeracil/oteracil for 3 cycles	No	CPS ≥ 5	NA
Kelly 2024 [17]	32	65 (39–73)	12 (37.5%)	NACT + CR	Nivolumab followed by CRT (Arm A; *n* = 15) vs. Nivolumab + Relatlimab followed by CRT (Arm B; *n* = 14)	Yes	CPS ≥ 1	22C3
Li 2023 [18]	25	63 (48–70)	25 (100%)	NACT	Camrelizumab (anti-PD1), Apatinib, and chemotherapy (S-1 ± Oxaliplatin) for 2–6 cycles	Yes	CPS ≥ 1	22C3
Ribeiro 2022 [22]	155	58.4 (12.5) (mean age + SD)	150 (96.77%)	NACT	Platinum-based doublets, Epirubicin-based triplets, or Taxane-based triplets.	No	CPS ≥ 1	22C3
Soeratram 2021 [23]	173	NA	NA	NACT + CR	CROSS regimen (Carboplatin + Paclitaxel)	No	CPS ≥ 1	28-8
Yu 2026 [24]	165	NA	NA	NACT	SOX/XELOX/other ± Anti-HER2 (Arm A, chemotherapy only, *n* = 49); Chemotherapy + PD-1 inhibitors/PD-L1 inhibitors/ PD-1/CTLA-4 bispecific ± Anti-HER2 (Arm 2, chemo-immunotherapy, *n* = 116)	Yes	CPS ≥ 1	22C3

NA—not available; NACT—neoadjuvant chemotherapy; CR—chemoradiation; SD—standard deviation.

**Table 3:** Baseline Clinical and Demographic Characteristics of Patients with Metastatic Disease.

Study (Author, Year)	Sample Size (*N*)	Age (Median/Range)	Therapeutic Regimen/Interventions	Anti PD-1 Treatment	Biomarker Assay; CPS Cut-Off;	PD-L1 Antibody Clone
Lim 2025 [19]	16	62 (36–76)	Cisplatin/Capecitabine/Trastuzumab (XPH)/5-FU; Pembrolizumab from 2 cycles	Yes	IHC; CPS ≥ 1	22C3
Massa 2024 [25]	22	65 (37–81)	Arm 1: Chemotherapy only (FOLFOX/XELOX) 16 patients (81.8%); Arm 2: Nivolumab + chemotherapy 4 patients (18.2%)	Yes	IHC; CPS ≥ 1	SP263
Chong 2024 [20]	72	NA	Anti-HER2 + anti-PD-1 and cisplatin or paclitaxel chemotherapy	Yes	SE-iFISH on CTC *; ≥10%	PD-L1/CD31-iFISH
Zhou 2020 [15]	189	60 (21–85)	NA	NA	IHC; CPS ≥ 1	22C3
Yang 2018 [16]	72	59 (21–84)	XELOX or FOLFOX for HER2-; Trastuzumab + 5-FU/Capecitabine + Cisplatin for HER2+;	No	IHC; NA	E1L3N

NA—not available; SE-iFISH—Subtraction enrichment-iFISH; CTC—circulating tumor cells; IHC—immunohistochemistry; * PD-L1 status was evaluated on circulating tumor cells (CTCs) via SE-iFISH using peripheral blood samples, in contrast to baseline tissue core biopsies/resections used in other studies.

**Table 4:** Pathological Responses and Survival Outcomes in the Neoadjuvant Treatment Cohort.

Study (Author, Year)	Total Efficacy Cohort (*n*)	Pathological Complete Response (pCR), *n* (%)	Pre-Treatment PD-L1+ in pCR Group, *n* (%)	Disease-Free Survival (DFS)	Median Follow-Up (Months)
Zhao 2024 [21]	104	4 (3.84%)	NA	NA	NA
Kelly 2024 [17]	29	9 (31.03%) (Arm A = 6/15 (40%); Arm B = 3/14 (21.42%))	7 (77.78%)	41.1 (29.4–NR)	36.4 (29.9–43.9)
Li 2023 [18]	19	3 (15.79%)	3 (100%)	NA	24.7 (20.9–31.8)
Ribeiro 2022 [22]	155	15 (9.68%)	NA	5-year DFS of 60.5%	60.3 (54.7–65.8)
Soeratram 2021 [23]	173	NA	NA	NA	NA
Yu 2026 [24]	162	29 (17.90%) (Arm A = 4/48 (8.3%); Arm B = 25/114 (21.9%))	NA	NA	NA

NA—not available; NR—not reached.

**Table 5:** Pathological Responses and Survival Outcomes in the Metastatic Cohort.

Study (Author, Year)	Total Efficacy Cohort (*n*)	Complete Response (CR), *n* (%)	Partial Response (PR), *n* (%)	Pre-Treatment PD-L1+ in PR, *n* (%)	Progressive Disease (PD), *n* (%)	Pre-Treatment PD-L1+ in PD, *n* (%)	ORR, % (95% CI/Range)	Median PFS (Months)
Lim 2025 [19]	16	0 (0%)	11 (68.75%)	11 (100%)	2 (12.5%)	1 (50%)	68.75% (54.3–95.9)	11.9 (5.12–18.28)
Massa 2024 [25]	22	NA	NA	NA	NA	NA	NA	NA
Chong 2024 [20]	72	NA	43 (59.72%)	NA	11 (15.27%)	NA	NA	7.03
Zhou 2020 [15]	189	NA	NA	NA	NA	NA	NA	NA
Yang 2018 [16]	72	3 (4.16%)	32 (44.44%)	20 (62.5%)	15 (20.83%)	6 (40%)	48.61%	6.4

NA—not available; PD—progressive disease; ORR—objective response rate; PFS—progression-free survival.

## Data Availability

Publicly available datasets were analyzed in this study. This data can be found in the article’s main text and reference list.

## Supplementary Materials

The following supporting information can be downloaded at https://www.mdpi.com/article/10.3390/cancers18183065/s1, Table S1. PRISMA 2020 Checklist [45]; Table S2: Methodological quality and risk of bias assessment of included studies using the Newcastle–Ottawa Scale (NOS); Table S3: Summary of PD-L1 Positivity Rates Before and After Treatment Across Included Studies; Table S4: Treatment-Induced PD-L1 Gain and Loss in Paired Biopsies.
