# Supplementary material for: Treatment-Induced PD-L1 Dynamics and Temporal Heterogeneity in Locally Advanced and Metastatic Gastroesophageal Cancer—A Systematic Review
Source: Cancers (Basel). 2026 Sep 21;18(18):3065. doi: 10.3390/cancers18183065 (PMC13605973; doi:10.3390/cancers18183065)
Supplement: Supplementary file 1 [file cancers-18-03065-s001.zip › Tables S2-S4.pdf]

Supplementary Materials

Treatment-Induced PD-L1 Dynamics and Temporal Heterogeneity in locally advanced and metastatic Gastroesophageal Cancer — A Systematic Review

Table S2. Methodological quality and risk of bias assessment of included studies using the Newcastle–Ottawa Scale (NOS).

Study (Author, Year)	Selection (max 4 points)	Comparability (max 2 points)	Outcome (max 3 points)	Total Score (0–9)	Risk of Bias Category
Zhao 2024 [17]	4	2	3	9	Low Risk (High Quality)
Kelly 2024 [18]	4	2	2	8	Low Risk (High Quality)
Li 2023 [19]	4	2	2	8	Low Risk (High Quality)
Ribeiro 2022 [20]	4	2	2	8	Low Risk (High Quality)
Soeratram 2021 [21]	3	2	1	6	Moderate Risk
Yu 2026 [22]	4	2	1	7	Low Risk (High Quality)
Lim 2025 [23]	4	2	2	8	Low Risk (High Quality)
Massa 2024 [24]	4	2	3	9	Low Risk (High Quality)
Chong 2024 [25]	4	2	2	8	Low Risk (High Quality)
Zhou 2020 [15]	3	2	3	8	Low Risk (High Quality)
Yang 2018 [16]	4	2	3	9	Low Risk (High Quality)

Note: Scores are assigned based on the adapted Newcastle-Ottawa Scale for cohort/paired non-randomized studies (0–9 points). A total score of ≥ 7 indicates low risk of bias (high quality), 4–6 indicates moderate risk, and ≤ 3 indicates high risk of bias.

Scoring criteria for paired PD-L1 evaluation studies: Selection (S1–S4): S1 — representative cohort, S2 — internal baseline control (paired biopsy), S3 — documented treatment records, S4 — pre-treatment PD-L1 status. Comparability (C1–C2): C1 — identical IHC assay/clone and cutoff pre/post-treatment, C2 — standardized clinical sampling method. Outcome (O1–O3): O1 — blinded pathologist assessment, O2 — adequate time interval between biopsies, O3 — completeness of paired tissue (<10% missing data).

Table S3. Summary of PD-L1 Positivity Rates Before and After Treatment Across Included Studies.

**Study (Author, Year)**	**Pre-treatment *N***	**Pre-treatment Positive, *n* (%)**	**Pre-treatment 95% CI (%)**	**Post-treatment *N***	**Post-treatment Positive, *n* (%)**	**Post-treatment 95% CI (%)**
Zhao 2024 [17]	104	32 (30.8%)	22.1 – 40.6	104	51 (49.0%)	39.1 – 59.0
Kelly 2024 [18]	14	9 (64.3%)	35.1 – 87.2	14	11 (78.6%)	49.2 – 95.3
Li 2023 [19]	18	3 (16.7%)	3.6 – 41.4	18	6 (33.3%)	13.3 – 59.0
Ribeiro 2022 [20]	74	4 (5.4%)	1.5 – 13.3	74	16 (21.6%)	12.9 – 32.7
Soeratram 2021 [21]	173	16 (9.2%)	5.4 – 14.6	119	11 (9.2%)	4.7 – 15.9
Yu 2026 [22]	109	82 (75.2%)	66.0 – 83.0	109	91 (83.5%)	75.2 – 89.9
Lim 2025 [23]	9	8 (88.9%)	51.8 – 99.7	9	6 (66.7%)	29.9 – 92.5
Massa 2024 [24]	9	6 (66.7%)	29.9 – 92.5	9	4 (44.4%)	13.7 – 78.8
Chong 2024 [25]	18	5 (27.8%)	9.7 – 53.5	18	4 (22.2%)	6.4 – 47.6
Zhou 2020 [15]	83	45 (54.2%)	42.9 – 65.2	83	44 (53.0%)	41.7 – 64.1
Yang 2018 [16]	72	42 (58.3%)	46.1 – 69.8	72	30 (41.7%)	30.2 – 53.9

Notes: N — total evaluated patients; n — number of PD-L1 positive cases; CI — confidence interval calculated using the Clopper-Pearson exact binomial method.

This table summarizes the baseline (pre-treatment) and post-treatment PD-L1 positivity rates reported across all included studies, stratified by treatment setting (neoadjuvant vs. palliative). Proportions are presented as raw patient counts (*n*), percentages (%), and corresponding exact 95% confidence intervals (95% CIs).

Table S4. Treatment-Induced PD-L1 Gain and Loss in Paired Biopsies.

Study (Author, Year)	Paired *N*	PD-L1 Gain, *n* (%)	Gain 95% CI (%)	PD-L1 Loss, *n* (%)	Loss 95% CI (%)	McNemar p-value
Kelly 2024 [18]	14	2 (14.3%)	1.8 – 42.8	0 (0.0%)	0.0 – 23.2	0.500
Li 2023 [19]	18	6 (33.3%)	13.3 – 59.0	0 (0.0%)	0.0 – 18.5	**0.031**
Ribeiro 2022 [20]	74	14 (18.9%)	10.7 – 29.7	2 (2.7%)	0.3 – 9.4	**0.004**
Soeratram 2021 [21]	111	7 (6.3%)	2.6 – 12.6	8 (7.2%)	3.2 – 13.7	1.000
Yu 2026 [22]	109	19 (17.4%)	10.8 – 25.9	10 (9.2%)	4.5 – 16.2	0.136
Lim 2025 [23]	9	0 (0.0%)	0.0 – 33.6	2 (22.2%)	2.8 – 60.0	0.500
Massa 2024 [24]	9	2 (22.2%)	2.8 – 60.0	4 (44.4%)	13.7 – 78.8	0.687
Chong 2024 [25]	18	4 (22.2%)	6.4 – 47.6	5 (27.8%)	9.7 – 53.5	1.000
Zhou 2020 [15]	83	15 (18.1%)	10.5 – 28.0	16 (19.3%)	11.4 – 29.4	1.000
Yang 2018 [16]	72	12 (16.7%)	8.9 – 27.3	24 (33.3%)	22.7 – 45.4	0.065

Notes: Paired *N* — number of patients with available paired pre- and post-treatment tissue samples; *n* — number of patients experiencing biomarker shift; CI — confidence interval calculated using the Clopper-Pearson exact binomial method. McNemar p-value was derived from exact binomial testing on discordant pairs (gain vs. loss). Bold values indicate statistical significance ($p < 0.05$).

This table provides detailed patient-level status shift data for paired baseline and post-treatment biopsy samples. PD-L1 appearance (gain) represents patients who converted from PD-L1 negative at baseline to PD-L1 positive post-treatment. PD-L1 loss represents patients who lost PD-L1 expression following therapy. Rates are presented with corresponding 95% CIs. Statistical significance of discordant shifts (gain vs. loss) was assessed using McNemar's exact test.
